# New Trends in Sexually Transmitted Infections Among Adolescents and Young People: Epidemiology, Clinical and Diagnostic Management

**DOI:** 10.3390/microorganisms13061411

**Published:** 2025-06-17

**Authors:** Nunzia Zanotta, Nicole West, Carolina Cason, Stefania degli Ivanissevich, Alessia Meneghel, Francesco Righi, Laura Brunelli, Alessandra Casuccio, Silvia Gazzetta, Daniele Gianfrilli, Teresa Maria Assunta Fasciana, Maria Cristina Salfa, Franz Sesti, Barbara Suligoi, Francesca Valent, ESPRIT Study Collaboration Group, Manola Comar

**Affiliations:** 1 Department of Advanced Translational Microbiology, Institute for Maternal and Child Health-IRCCS Burlo Garofolo, 34137 Trieste, Italy; nunzia.zanotta@burlo.trieste.it (N.Z.);; 2 Department of Medical, Surgical, and Health Sciences, University of Trieste, 34127 Trieste, Italy; nicolewest.tlb@gmail.com (N.W.);; 3 Department of Medicine, University of Udine, 33100 Udine, Italy; 4 SOC Accreditation, Quality, and Clinical Risk, University Health Authority Friuli Centrale, 33100 Udine, Italy; 5 Section of Hygiene, PROMISE Department, University of Palermo, 90127 Palermo, Italy; alessandra.casuccio@unipa.it; 6 Department of Experimental Medicine, Sapienza University of Rome, 00185 Rome, Italyfranz.sesti@uniroma1.it (F.S.); 7 Department of Health Promotion, Mother and Child Care, Internal Medicine and Medical Specialities, University of Palermo, 90127 Palermo, Italy; teresa.fasciana@unipa.it; 8 AIDS Operational Centre, Department of Infectious Diseases, Istituto Superiore di Sanità, 00161 Rome, Italy; mariacristina.salfa@iss.it (M.C.S.);; 9 SOC Hygiene and Public Health, Department of Prevention, University Health Authority Friuli Centrale, 33100 Udine, Italy; francesca.valent@asufc.sanita.fvg.it

**Keywords:** epidemiology, adolescents, sexually transmitted infections, public health, prevention strategies, clinical and laboratory management, narrative review

## Abstract

Sexually transmitted infections (STIs) are a significant public health issue, especially among adolescents and young adults. Despite improvements in diagnostic tools and treatments, over 1 million new STIs occur daily worldwide, many of which are asymptomatic. These infections can severely affect quality of life and reproductive health, particularly when contracted at a young age. This review provides an overview of STIs’ recent epidemiology data, clinical trends, and diagnostic challenges in Italian adolescents and young adults, focusing on the *Chlamydia trachomatis*, *Neisseria gonorrhoeae*, *Treponema pallidum*, *Thricomonas vaginalis*, and *Mycoplasma*/*Ureaplasma* species. Worrying new evidence indicates that young women are at a higher risk of contracting STIs than men and multidrug-resistant strains have increased in young heterosexuals. This evidence shows a general change in lifestyle, where a lack of awareness about the risks of STI reflects a significant educational gap. To address the rising STI rates, targeted school educational interventions and innovative multidisciplinary healthcare models, such as the hub-and-spoke approach, are needed.

## 1. Introduction

In young people and adolescents, sexually transmitted infections (STIs) remain a major focus within the field of sexual health, despite the availability of advanced diagnostic tools and therapies. STIs refer to a wide range of bacterial, fungal, viral, and protozoal infections, most of which are asymptomatic and share a common mode of transmission through unprotected sexual intercourse [1]. These infections are primarily caused by pathogens such as *Chlamydia trachomatis*, *Neisseria gonorrhoeae*, *Treponema pallidum*, and *Trichomonas vaginalis*, as well as viral agents such as Herpes Simplex Virus, Papillomavirus, HCV, and HIV. Their symptoms can affect quality of life and significantly impact both individual reproductive health and sustainability of the healthcare system. Some STIs, including infection caused by *Mycoplasma*/*Ureaplasma* species, are often overlooked due to the perception that these microorganisms are merely bystanders and not directly involved in the pathogenetic process. However, aside from *Mycoplasma genitalium* [2], recent data show an increased association between other species of this family and urogenital symptoms or mixed infections, a trend that is particularly evident among younger generations [3].

In 2023, the European Centre for Disease Prevention and Control (ECDC) reported that *Chlamydia trachomatis* remains, up to now, the leading bacterial cause of STIs, with young people aged 15–24 accounting for 68% of total cases [4]. At the same time, a concerning increase in *Neisseria gonorrhoeae* cases was reported among heterosexual young people [5].

In Italy, surveillance system for sexually transmitted diseases is managed by the Istituto Superiore di Sanità (ISS) through a sentinel network of highly specialized public clinical centers, active since 1991, which reports new STI cases among symptomatic patients, collecting socio-demographic, behavioral, and clinical data. Since 2009, the system has also included clinical microbiology laboratories that report individuals undergoing laboratory tests for *Chlamydia trachomatis*, *Trichomonas vaginalis*, and/or *Neisseria gonorrhoeae*, regardless of the presence of symptoms. Data are collected by the centers and submitted through an online web-based reporting system.

Data collected from Sentinel Surveillance Systems covering the period 2020–2022 showed an overall increase in STIs of approximately 18%. *Neisseria gonorrhoeae*, *Treponema pallidum*, and *Chlamydia trachomatis* were the most frequently detected microorganisms. Among young people, the prevalence of *Chlamydia trachomatis* was estimated to be three times higher compared to older individuals, and young women were at higher risk of contracting STIs than men [6,7]. These data indicate that adolescents and young adults are particularly vulnerable to STIs, representing a new silent cluster in the spread of these infections. Although influenced by various socioeconomic and cultural factors, this evidence reflects a general change in lifestyle, where a lack of awareness about the risks of STI transmission reveals a significant educational gap. In particular, adolescents show limited knowledge and risk perception [8,9], and consequently, the burden of STIs remains alarming, representing one of the top 20 global causes of DALY (Disability Adjusted Life Years) in individuals under 19 years of age [10]. The control of STIs has become a public health priority. New strategies must be adopted, including innovative, professional, multidisciplinary models with high quality STI care (hub-STI centers) and regional support structures (spoke-STI services) [11], together with active interventions in educational and formative programs involving school and parents [12,13].

In particular, the implementation of pediatric STI services presents distinct challenges that differentiate them from adult-focused initiatives. Many existing healthcare centers and infection management protocols are primarily designed for adults, highlighting the need for context-specific strategies to ensure the effective involvement of adolescents and young people, considering patient complexity and the parent–child dynamic. Emerging data among young people indicate the spread of multidrug-resistant STI strains resulting from genetic selective pressure and the indiscriminate use of antibiotics. For *Neisseria gonorrhoeae*, the second most common bacterial STI in Europe, the emergence of resistance to last-line therapies, such as ceftriaxone, has become a major public health concern due to the limited available therapeutic alternatives and the lack of a vaccine [14,15]. Moreover, untreatable infections caused by *Mycoplasma genitalium* have already been reported. On the other hand, *Chlamydia trachomatis* and *Treponema pallidum* remain susceptible to first-line therapies as follows: tetracyclines and macrolides for *Chlamydia trachomatis*, and penicillin for *Treponema pallidum* [16].

This review aims to provide an overview of the epidemiological framework, clinical trends, and laboratory challenges associated with the most common treatable STIs among young people in Italy, including *Chlamydia trachomatis*, *Neisseria gonorrhoeae*, *Treponema pallidum*, and *Trichomonas vaginalis*. A dedicated section also focuses on emerging urogenital bacteria such as *Mycoplasmas* and *Ureaplasma* spp.

## 2. Methods

This narrative review focused on studies published between 2020 and 2024 that provide epidemiological data, clinical symptoms, diagnostic tests, and treatment protocols for the following sexually transmitted infections (STIs): *Chlamydia trachomatis*, *Neisseria gonorrhoeae*, *Treponema pallidum* (syphilis), *Trichomonas vaginalis*, and *Mycoplasma* species. A literature search was conducted in databases including PubMed, Scopus, and the Italian National Surveillance System (coordinated by the Italian Institute of Health) for relevant original studies published in English and Italian between 1 January 2020 and 31 December 2024. Eligible studies included peer-reviewed articles, clinical guidelines, and surveillance reports related to the prevalence, diagnosis, and management of STIs in adolescent and young adult populations. The search terms selected were epidemiology, clinical management, or diagnostic approaches for the mentioned microorganisms among adolescents or young adults. Exclusion criteria were studies with insufficient data on the target microorganisms and articles not available in full text. For each included study, data on the following were extracted:

Epidemiological data: Prevalence, incidence rates, and demographic distribution of STIs.

Clinical symptoms: Common symptoms associated with each STI.

Diagnostic tests: Methods used for diagnosis, including laboratory techniques and molecular tests.

Treatment protocols: First-line and alternative treatment options for each microorganism.

A qualitative synthesis was performed to summarize the findings from the included studies and presented narratively. Given the heterogeneity in study designs and methodologies, a meta-analysis was not conducted.

This review selected 136 studies that focused on the epidemiology of target microorganisms such as *Chlamydia trachomatis*, *Neisseria gonorrhoeae*, *Treponema pallidum* (syphilis), *Trichomonas vaginalis*, and *Mycoplasma* species at the global, European, and Italian levels. Regarding diagnostic tests and treatment, reference was made to studies based on Italian guidelines.

## 3. *Chlamydia trachomatis*

### 3.1. Epidemiology

*Chlamydia trachomatis* (CT) is the most commonly diagnosed bacterial STIs worldwide [17], with approximately 129 million new cases occurring each year among adults aged 15 to 49 years [18]. The global prevalence in this age group was estimated at 4.0% for women and 2.5% for men in 2020 [19], while the global incidence rate was 36 per 1000 women and 29 per 1000 men [20].

The Centers for Disease Control and Prevention (CDC) provided surveillance data for chlamydia infections in 2023, reporting that over 1.6 million cases of chlamydia were diagnosed in the United States, with the reported rate of chlamydia increasing slightly among men (1.3%) and decreasing among women (1.7%). Notably, 55.8% of all reported CT cases affected individuals aged 15 to 24, with a positive trend observed in heterosexual subjects. Nevertheless, the global prevalence was lower than that recorded in 2019.

In the United States, notification rates appear to have been influenced by lower adherence to screening programs following the COVID-19 pandemic [21].

In contrast, the European context is shaped by several factors beyond the pandemic, including the low availability of molecular diagnostics, the different characteristics of surveillance systems, the effectiveness national testing policies, and their levels of implementation [22].

Despite these challenges, record-high notification rates for 2022 were observed in both women and men. Young adults aged 20–24 had the highest age-specific notification rates, followed by those aged 15–19. In 2022, women in the 20–24 age group saw an 18% increase in rates compared to 2021, while men aged 25 and older had higher rates [22].

The most recent epidemiological data from the ECDC technical report (published in September 2024) highlight that sexually active young people aged 15 to 24, especially women, continued to exhibit the highest rates of reported CT infection in 2022. Specifically, among young people aged 15 to 24 years, CT prevalence is estimated to be 5.54% in young women and 3.32% in young men [23].

The Italian epidemiological data, updated to 2021 and published by the National Surveillance System coordinated by the Italian Institute of Health, highlighted an increase in CT prevalence among individuals aged 15–24 years compared to those over 24 years old (8.2% vs. 2.6%). Specifically, the prevalence of CT decreased with age, from 8.2% among individuals aged 15–24 years to 3.9% among those aged 25–34 years, and 1.8% among individuals older than 34 years. This decrease in CT prevalence by age group was observed in both women and men [24]. The data recorded by the ISS surveillance continued to show an increase in STIs, with a 25% rise in reported CT infection cases since 2019. Young people under 25 remain the most affected group, with a 7% prevalence in this demographic, compared to just 1% among individuals over 40 [24,25].

### 3.2. Clinical Manifestations

CT is a Gram-negative bacterium that infects genital tissues and is transmitted through vaginal, anal, or oral sex. It primarily affects epithelial cells in the male and female reproductive systems. The infection is often asymptomatic, with 40% to 96% of cases showing no symptoms. In women, symptoms may include yellowish vaginal discharge, bleeding, pain during sex or urination, and pelvic pain. If untreated, CT can cause more severe conditions like endometritis and salpingitis, and it is strongly linked to pelvic inflammatory disease (PID), infertility, and ectopic pregnancy [26].

It can also lead to miscarriages and preterm births, especially if acquired at 24 weeks of pregnancy [27,28,29]. Neonates may contract conjunctivitis or pneumonia during childbirth. CT infection has been associated with an increased risk of cervical cancer due to its presence in HPV lesions [30].

In men, CT can cause epididymitis, proctitis, and prostatitis, and it is the most common cause of nongonococcal urethritis [27,31]. It can also affect sperm motility and viability, contributing to male infertility [32]. Rectal and pharyngeal infections are typically asymptomatic, though rare cases show mucopurulent exudate and edema. Lymphogranuloma venereum (LGV), caused by CT L1-L3 serovars, is a common cause of severe proctitis [33,34,35]. Rectal infections are also seen in women and are not always linked to receptive anal intercourse, suggesting that genital infections could spread to the rectum. Persistent rectal infections may reinfect the genital tract. Genetic factors, such as mutations in the DEFB-126 gene, have been linked to increased susceptibility to CT infection [35,36,37].

### 3.3. Testing and Screening

Nucleic Acid Amplification Tests (NAATs) are the gold standard for CT diagnosis and screening due to their higher sensitivity (90–100%) and specificity (99–100%) compared to antigenic, hybridization, or culture methods [38,39]. The available laboratory platforms are combination assays capable of detecting, in real time, infections caused by *Neisseria gonorrhoeae*, *Chlamydia trachomatis*, *Trichomonas vaginalis*, and *Mycoplasma*/*Ureaplasma* in the same sample [39]. The samples to be analyzed are collected from the sites potentially infected by the bacteria, such as urogenital swabs, as well as oropharyngeal and rectal swabs for extragenital infection sites. Annual screening for chlamydia is recommended for all sexually active women under 25 years of age who have a new sexual partner, multiple partners, or a sexual partner with a current or recent STI (within the last six months); inconsistent condom use; engagement in commercial sex work; drug use; HIV infection with sexual activity; a previous chlamydia infection; or the presence of other STIs. The recommendation for annual chlamydia screening in women under 25 has been widely debated and discontinued in some countries. Critics argue it may not be cost-effective or supported by enough evidence for universal application, preferring targeted screening for high-risk individuals. On the other hand, there is insufficient evidence to recommend screening for heterosexual men who are at low risk for infection. However, for men who have sex with men (MSM), annual screening is recommended at sites of contact (urethra, rectum), regardless of condom use, and every 3 to 6 months for those at increased risk [40,41,42].

### 3.4. Treatment

Immediately after a positive CT test result, the patient must be treated, and treatment should also be provided to sexual partners to prevent reinfection and reduce the risk of further spread of the infection. According to the 2021 CDC guidelines, which are also confirmed by the most recent 2023 AMCLI guidelines (Association of Italian Clinical Microbiologists), recommended regimens for chlamydia infection among adolescents and adults include treatment with doxycycline 100 mg orally two times/day for 7 days. Alternative regimens involve azithromycin 1 g orally as a single dose or levofloxacin 500 mg orally once daily for 7 days. Doxycycline is preferred over other antibiotics, in particular, azithromycin, based on studies comparing the two molecules [38,41]. Data from randomized clinical trials have shown a higher rate of treatment failure among men treated with azithromycin compared to those treated with doxycycline [43,44]. Observational studies have also shown that doxycycline is more effective than azithromycin in treating rectal CT infection in both men [43] and women [45]. Abstinence from sexual intercourse is always recommended for up to 7 days after completing the treatment and, in any case, up to 7 days after the end of the partner’s treatment.

In men, if there is co-infection with CT and *Mycoplasma genitalium*, azithromycin is the first-line drug for 5 days, with a dose of 500 mg on the first day and 250 mg for the remaining 4 days.

First-line treatment for Lymphogranuloma Venereum (LGV) caused by infection with unique strains of CT is doxycycline for 21 days for asymptomatic individuals and for 7 days for symptomatic individuals who test positive on the diagnostic test. Finally, erythromycin and ciprofloxacin are the third-line treatment options. The initial drug is administered for a period of 10–14 days (500 mg twice a day), while the second is administered for 7 days (200 mg twice a day or a single dose of 400 mg). According to the 2021 CDC guidelines on CT infection during pregnancy, azithromycin is the treatment of choice [40]. They do not recommend doxycycline during pregnancy, particularly in the second half, as it can cause permanent tooth discoloration during fetal tooth development [46].

Currently, no chlamydia vaccine has been approved for the market but CTH522/CAF^®^01 is the first vaccine to have successfully completed Phase I clinical trials, demonstrating a good safety profile and providing data for further studies on how findings in mice may translate to humans [47].

## 4. *Treponema pallidum*

### 4.1. Epidemiology

Syphilis is an STI caused by the bacterium *Treponema pallidum* (TP). Although treatable, if left untreated, syphilis can cause severe health complications, including neurological and cardiovascular damage. Over the past two decades, syphilis incidence has significantly increased. In 2022, WHO member states set a goal to reduce syphilis infections by 2030, but new cases continue to rise [48,49]. The 2022 data showed a notable increase in cases, particularly in the 15–49 age group, with the highest surges in the Americas and African regions. In 2023, the CDC reported over 209,000 syphilis cases, including congenital syphilis, representing a 1% increase from 2022. Bisexual individuals and MSM (men who have sex with men) are disproportionately affected, often with co-occurring HIV infections [50,51]. The ECDC 2022 Syphilis Annual Epidemiological Report revealed a 34% increase in the crude notification rate compared to 2021 and a 41% increase since 2018, highlighting the ongoing public health challenge. Among the 26 countries reporting data, 11% of syphilis cases were in the 15–24 age group, with 9% in 20–24-year-olds and 2% in 15–19-year-olds. Men had higher age-specific rates than women, with men aged 20–24 showing 23 cases per 100,000 population, and men aged 25–34 showing 40 cases per 100,000. Notably, syphilis rates for men aged 20–24 increased by 41% between 2021 and 2022, while women aged 20–24 saw a 50% increase [52]. These trends suggest the need for targeted interventions for specific age and risk groups. In Italy, syphilis cases increased by 34% from 2021 to 2022. While diagnoses remain more common among young men, there has been a notable rise in cases among women in recent years. Since 2013, syphilis diagnoses in Italy have gradually increased, with a brief stabilization during the COVID-19 pandemic at over 150 cases per year. Current data emphasize the importance of ongoing surveillance and targeted interventions to address the evolving epidemiology of syphilis worldwide [24].

### 4.2. Clinical Manifestations

Syphilis is spread through direct contact during vaginal, anal, or oral intercourse, from mother to child during pregnancy, and though rarely, through blood. It progresses through four stages as follows: primary, secondary, latent, and tertiary, each with distinct symptoms. The incubation period between infection and primary symptoms ranges from 10 to 90 days [53,54,55]. The primary stage is marked by a chancre, a painless, hard, dark-red lesion that resolves in 3–6 weeks, but the infection continues. The secondary stage, occurring 3–12 weeks later, involves copper-colored rashes, often on the palms and soles, mucous membrane lesions, sore throat, and systemic symptoms like headache, fatigue, and gastrointestinal issues. Meningitis and cranial nerve palsies can occur in some cases, and painless lymphadenopathy affects 70–85% of patients. Malignant syphilis, a rare secondary form, involves severe lesions [55,56,57].

In the latent stage, which lasts up to two years, there are no symptoms, but the individual remains infectious. Latent syphilis may recur at an early or late stage, with a reduced risk of transmission in the latter. If untreated, syphilis progresses to tertiary syphilis, which can occur 10–30 years later and cause severe complications such as cardiovascular syphilis, neurosyphilis, and gummatous lesions affecting various organs. Cardiovascular syphilis may lead to aortic aneurysm and stroke, while neurosyphilis can result in cognitive and motor impairments and potentially fatal outcomes [53,58,59].

Congenital syphilis occurs when the infection is transmitted from an untreated mother to her fetus, potentially causing miscarriage, preterm birth, stillbirth, or birth defects. The severity of congenital syphilis depends on the timing of maternal infection during pregnancy [52,60].

In women, syphilis can lead to pelvic inflammatory disease, infertility, chronic pelvic pain, and complications during pregnancy such as ectopic pregnancy, miscarriage, and neonatal transmission. However, there is no substantial evidence linking syphilis to male infertility [49].

### 4.3. Testing and Screening

There are both indirect and direct methods for detecting TP. Direct methods include dark-field microscopy (DFM), direct fluorescence antibody (DFA) testing, immunohistochemistry (IHC), and NAATs, though commercially available NAATs for TP DNA are not yet available [61,62]. Some laboratories offer locally developed PCR tests for TP detection. TP cannot be cultured on routine media, and dark-field microscopy is mainly used in early syphilis when antibodies have not yet been produced. This method requires a trained technician and lesion exudate or tissue. Indirect serological tests are used the most, which search for antibodies against TP antigens (treponemal tests) or against lipoidal antigens (non-treponemal tests) [38,62,63,64,65]. Common treponemal tests include TPHA, TPPA, EIA/ELISA, CLIA/CMIA/ECLIA, FTA-ABS, and Western blot for IgG and IgM. Non-treponemal tests, such as RPR and VDRL, are less specific but help monitor disease progression [63,64]. However, false positives can occur, especially in cases involving autoimmune diseases, older age, or injecting drug use. Serological tests are used for diagnosis, screening asymptomatic individuals, confirming positive diagnoses, and monitoring treatment progress [38,66]. Screening is recommended for high-risk individuals, such as MSM, HIV-positive persons, and those with a history of incarceration or transactional sex work. In August 2024, the FDA approved the first self-directed rapid syphilis test for in-home diagnosis, although positive results must be confirmed by laboratory tests [41,67,68]

### 4.4. Treatment

Syphilis progresses through different stages, and treatment depends on the clinical manifestations at each stage. Penicillin G, administered parenterally, is the first-line treatment for all stages, with dosage and duration varying based on the clinical presentation. Longer treatment regimens are required for late latent syphilis, tertiary syphilis, and unknown timing of infection. The formulation of penicillin G’s is critical, as TP can persist in immune-privileged sites like the central nervous system. According to the 2021 CDC guidelines, the treatment for primary and secondary syphilis in adults is a single dose of 2.4 million units of benzathine penicillin G intramuscularly (IM), effective even in HIV-co-infected patients [40]. For neonates and children, a weight-based dose is recommended. In latent syphilis, early latent syphilis requires a single dose, while late latent syphilis needs three doses over three weeks. HIV co-infection does not alter these regimens [40]. For tertiary syphilis without neurological involvement, 7.2 million units of benzathine penicillin G are administered in three weekly doses [41]. If neurological complications like neurosyphilis are present, treatment with aqueous crystalline penicillin G (18–24 million units daily) is necessary for 10–14 days [41]. Pregnant women should follow the recommended penicillin regimen based on the infection stage, with no contraindications to its use [69]. The treatment for congenital syphilis varies based on clinical and laboratory findings in the newborn, with aqueous crystalline penicillin G being the main treatment for confirmed cases [41,70,71].

## 5. *Neisseria gonorrhoeae*

### 5.1. Epidemiology

Gonorrhoeae is the second most prevalent STI, caused by the bacterium *Neisseria gonorrhoeae* (NG). It is primarily transmitted through vaginal, oral, or anal sex. In 2020, the WHO estimated 82.4 million new infections globally among adults. Due to increasing drug resistance, gonococcal infection has become a serious public health issue over the years and is the focus of specific global initiatives. For this reason, the Global Health Sector Strategies on HIV, Viral Hepatitis, and Sexually Transmitted Diseases 2022–2030 have included NG in their program, aiming for a 90% reduction in gonorrhoeae incidence by 2030 [52].

Additionally, the WHO collaborates with countries to improve the management of antimicrobial resistance through the European Gonococcal Antimicrobial Surveillance Programme (Euro-GASP), which focuses on implementing surveillance systems to detect antimicrobial resistance in NG and guide appropriate local-level treatment [72]. The latest Gonorrhoeae Annual Epidemiological Report for 2022 documented [72] 881 confirmed cases of gonorrhoeae. This represents a 48% increase in crude notification rates compared to 2021 and a 59% increase from 2018. However, a decrease was observed in 2020 during the first year of the COVID 19 pandemic, attributed to changes in healthcare seeking behavior, disruptions in sexual health services, and a reduction in testing [73,74].

In 2022, a significant increase in gonorrhoeae notifications was observed among the youngest age groups, specifically those aged 15–19 and 20–24 years, in both women and men. These trends were also confirmed in early 2023 [22]. A recent systematic review by the ECDC reported that the overall prevalence of gonococcal infection was estimated to be 0.24% in women and 0.10% in men. Among young women, the prevalence was 0.51%, compared to 0.07% in young men [22].

In Italy, the highest increase in the reporting rate was observed in 2022, with a 63% rise compared to 2021. Specifically, the largest increase among women was observed in the 20–24 age group, compared to the male population, which saw a 50% increase in the same age group [24].

In the 15–19 age group, the most substantial increase in the notification rate was observed in the male population, compared to the female population, with increases of 57% and 49%, respectively [75]. However, this rise is concerning, particularly given the growing antibiotic resistance of NG, which has reached 22% for azithromycin in Italy [24].

### 5.2. Clinical Manifestations

NG is a Gram-negative diplococcus responsible for gonorrhea, a major global public health issue. NG primarily infects the urogenital tract, but can also colonize extragenital sites like the eyes, nasopharynx, and anus [76,77].

Transmission occurs through sexual fluids such as vaginal fluid and semen. Men are more likely to show symptoms than women, with symptoms in women often being nonspecific, such as odorless vaginal discharge and post-coital bleeding, which are frequently misdiagnosed as bacterial vaginosis or yeast infections [78,79]. Cervicitis, inflammation of the cervix, is a common sign of NG infection. If untreated, NG can cause severe reproductive complications, including endometritis, salpingitis, and pelvic inflammatory disease (PID). In rare cases, it can lead to bacteremia and disseminated infections. PID symptoms include lower abdominal pain, tenderness, and cervicovaginal inflammation, potentially resulting in infertility, ectopic pregnancy, and chronic pelvic pain [28,80]

In men, NG infections are often symptomatic, with urethral discharge, painful urination, and testicular pain or swelling being common. If untreated, complications like urethritis, epididymitis, prostatitis, proctitis, and reactive arthritis may occur [81]. NG may also cause sperm damage, potentially affecting fertility [82]. Anorectal infections are common in MSM and women with anal intercourse exposure. These infections are mostly asymptomatic but can cause anal itching, mucopurulent discharge, and discomfort [83,84]. Pharyngeal infections are often asymptomatic or cause mild symptoms, but they are harder to treat due to antimicrobial resistance [85].

NG can also cause gonococcal ophthalmia neonatorum, a severe conjunctivitis in newborns exposed to infected cervical secretions during birth. Immediate treatment is essential to prevent complications like corneal scarring and blindness [86,87].

### 5.3. Testing and Screening

NAATs are the most sensitive diagnostic tools for detecting NG, with sensitivity exceeding 90% and specificity above 98% for both genital and extragenital sites. They have a higher sensitivity than the culture and the test is not influenced by the transport and storage of the sample. However, despite their advantages, commercial NAATs do not provide information on antimicrobial susceptibility, making parallel culture testing essential for comprehensive diagnostic workflows. Positive culture on Thayer Martin Agar and Chocolate Agar plates for 24–48 h, showed small convex, gray, and shiny colonies with a diameter of up to 1 mm appear. The identification of the bacterial species is carried out by at least two tests as follows: the biochemical test (API NH, VitekII) and MALDI-TOF (matrix-assisted laser desorption ionization time-of-flight) mass spectrometry. Growth and isolation in culture is the only method to assess sensitivity to antimicrobials such as ceftriaxone, azithromycin, cefixime, and ciprofloxacin [38,88].

EUCAST does not define a reference method for determining the MIC of these molecules and does not define clinical breakpoints for azithromycin, which are provided by the CLSI Publishes M100-Performance Standards for Antimicrobial Susceptibility Testing, 32nd Edition, 2022 [38].

Other diagnostic methods, such as DNA probe assays, antigen detection tests, and serology for antibodies against NG, are generally not recommended due to their insufficient sensitivity and specificity [88]. Specimen collection varies depending on the patient population and infection site. In women, vaginal swabs are the preferred specimens for NG detection; however, endocervical swabs and first-catch urine samples are also acceptable. In men, first-catch urine or urethral swabs are preferred for urogenital infections, while for extragenital infections, rectal or pharyngeal swabs are required for both women and men [61]. Gonococcal infections are often asymptomatic at both genital and extragenital sites, such as anorectal and oropharyngeal areas. These asymptomatic cases represent significant reservoirs for transmission. To date, annual screening is recommended for all sexually active women under 25 years of age who meet risk criteria, including new or multiple sexual partners, inconsistent condom use, commercial sex work, drug use, a history of STIs, or HIV infection [41,61,89]. Similarly, sexually active men and transgender women who have sex with men are advised to undergo annual genital and rectal screening [61].

### 5.4. Treatment

The latest updates on the treatment of gonococcal infections were published by the WHO in July 2024, covering adults, adolescents, pregnant women and HIV-positive patients. With rising antimicrobial resistance in NG, reduced drug efficacy, and the absence of an effective vaccine, treatment decisions must be informed by national or local antimicrobial resistance data [90].

The 2024 WHO guidelines now recommend a single IM dose of ceftriaxone 1 g for all patients with genital, anorectal, and/or oropharyngeal infections. If ceftriaxone is unavailable or declined, cefixime 800 mg orally is an alternative, provided a test of cure is conducted. In cases where a test of cure is not feasible or oropharyngeal infections are present, cefixime 800 mg orally combined with azithromycin 2 g orally is advised. The emergence of strains resistant to ceftriaxone and azithromycin has undermined first-line treatment options [90,91,92]. For patients with cephalosporin resistance, allergy, or limited availability, spectinomycin 2 g IM combined with azithromycin 2 g orally is recommended. Alternatively, gentamicin 240 mg IM plus azithromycin 2 g orally can be used. For treatment failures in adults and adolescents, including pregnant women, the WHO advises cefixime 800 mg IM plus azithromycin 2 g orally (if ceftriaxone was not previously administered), spectinomycin 2 g IM plus azithromycin 2 g orally, or gentamicin 240 mg IM plus azithromycin 2 g orally [90,91]. Following the conclusion of the treatment regime, it is recommended that a culture test be performed. In the case of a positive result, it is advisable to administer a single oral dose of 800 mg of cefixime. Two alternative second-line treatments are ceftriaxone, administered as a single intramuscular dose of 500 mg, and gentamicin and azithromycin, administered as a single intramuscular dose of 240 mg for the former and 2 g for the latter, with the latter being administered orally [39].

## 6. *Trichomonas vaginalis*

### 6.1. Epidemiology

*Trichomonas vaginalis* (TV) is a sexually transmitted protozoon that infects the urogenital tract. TV is estimated to be the most common non-viral STI worldwide. According to the latest data from the WHO, the global incidence of TV infection in 2016 was estimated at 156 million cases (73.7 million females and 82.6 million males) among people aged 15 to 49 years. The most recent incidence data estimate that the number of new TV infections was nearly 7 million (3.3 million men and 3.5 million women). The 15–24 age group accounted for 16.3% of incident infections (568,000 men vs. 520,000 women) [93]. However, these epidemiological data may be underestimated due to the high number of asymptomatic cases [94]. In Europe, the most recent technical report from the ECDC estimated a prevalence of 0.69% among women and 0.00% among men [22]. However, in most European countries, routine screening programs for STIs do not include TV [95]. In Italy, TV surveillance has been introduced in recent years, and data from the Sentinel Surveillance System, as of 2021, report a prevalence of 1% (1.1% among men and 1.2% among women) [24].

### 6.2. Clinical Manifestations

Trichomonas vaginalis (TV) exclusively infects human cells and is primarily transmitted through sexual intercourse. Evidence shows a higher infection rate among male partners of infected women, as well as increased prevalence in women attending Sexually Transmitted Disease (STD) clinics or sex workers [96,97]. TV typically infects the squamous epithelium of the genital tract, with an incubation period ranging from 4 to 28 days. The infection can persist for long durations in women but usually resolves more quickly in men [96].

In women, TV is symptomatic in about 50% of cases, with around 30% of initially asymptomatic cases becoming symptomatic within six months. Common symptoms include yellow–green, frothy, fishy-smelling vaginal discharge; irritation; soreness; pain during sex; and swelling in severe cases. Painful or frequent urination is also common, and vaginal and urinary symptoms may occur together. Acute cases may show hemorrhagic spots on the vaginal and cervical mucosa, known as “strawberry cervix” [98]. If untreated, TV can lead to complications like cervicitis, urethritis, Pelvic Inflammatory Diseases (PID) cervical intraepithelial neoplasia, and adverse reproductive outcomes such as preterm labor and low birth weight, especially in those co-infected with HIV [99,100,101].

In men, TV is mostly asymptomatic, but when symptoms occur, they include frothy penile discharge, painful urination, and frequent urges to urinate. TV can cause urethritis, prostatitis, and epididymitis, and chronic infection of the prostate may increase the risk of prostate cancer [102]. Neonatal trichomoniasis can occur during birth, typically presenting as a vaginal infection, and in rare cases, neonates may develop respiratory issues due to TV [103,104].

TV infection also significantly impacts women’s reproductive health, with links to infertility. Key mechanisms include compromised sperm quality, immune system activation causing inflammation, damage to oocytes, and ovulation blockage [105,106].

### 6.3. Testing and Screening

The most common rapid and inexpensive method for diagnosing TV is wet prep microscopy. This can be performed in a clinical setting as point-of-care testing, but it has a low sensitivity of 60–70% in women and 30% in men. The sample must be analyzed within a few minutes of collection to avoid false-negative results. TV is a motile organism with flagella and can be seen moving in the preparation when viewed under a microscope. The most reliable specimens for diagnosing female trichomoniasis include endocervical and vaginal swabs, as well as urine; while in male patients, the reliable specimens are urine, urethral swabs, and semen [94,107].

Liquid or broth culture of a clinical specimen (cervicovaginal, urethral, or urinary sediment) for microscopic observation was previously the gold standard technique for the diagnosis of trichomoniasis, due to its sensitivity, simplicity, and the relatively low inoculum requirement (300 trichomonas/mL) [94]. Molecular techniques are considered the appropriate techniques for the diagnosis of TV, these tests can detect the genome of the parasite in symptomatic and asymptomatic patients, with a high sensitivity (97.2%) and specificity (99.9%). Molecular tests can be performed on vaginal, endocervical, or urine samples in women and on urethral or urine samples in men [37]. Annual screening is recommended for women receiving care in high-prevalence settings (e.g., STI clinics and correctional facilities) and for asymptomatic women at high risk for infection (e.g., women with multiple sexual partners, exchanging sex for payment, drug use, or a history of STIs or incarceration). Screening is also recommended for sexually active women with HIV at the time of entry to care and at least annually thereafter [41,107].

### 6.4. Treatment

Trichomoniasis is unlikely to resolve spontaneously without treatment, and it is typically treated effectively and efficiently with antibiotics. According to the latest guidelines from the WHO, the treatment protocol is consistent across adults, adolescents, pregnant women, and individuals co-infected with HIV. The recommended therapy involves metronidazole at a dosage of 400 mg or 500 mg orally twice daily for 7 days. Tinidazole is another effective treatment for trichomoniasis, with the advantage of fewer side effects compared to metronidazole. However, for cases of metronidazole-resistant infections, tinidazole is typically ineffective due to the structural similarities between the two molecules [108,109,110,111]. Despite being more expensive than metronidazole, tinidazole offers a longer half-life (12.5 h vs. 7.3 h for metronidazole) and causes fewer gastrointestinal side effects. In cases where metronidazole or tinidazole is unavailable, secnidazole or ornidazole may serve as alternatives, though both are contraindicated in pregnancy [108]. Similarly, ornidazole has demonstrated efficacy and safety, with the advantage of single-dose administration.

To prevent reinfection, it is crucial to treat both the patient and all sexual partners. Sexual activity should be avoided until all individuals have completed treatment and symptoms have been resolved. This is essential because TV infection does not confer immunity, leaving individuals susceptible to reinfection. Common causes of recurrence include non-adherence to therapy, treatment failure, or reinfection from an untreated partner [41].

HIV and TV co-infection are significant public health concerns. Studies reveal that approximately half of HIV-positive women also have TV infection. Effective treatment of TV not only resolves the infection but may also reduce the risk of HIV transmission and improve health outcomes for co-infected individuals [41]. The most recent AMCLI guidelines recommend that for the treatment of male urethritis and cervicitis, the preferred option is the one outlined by the CDC [41]. In addition, they suggest a second-line treatment option, which is the oral administration of tinidazole in a single 2 g dose [38].

## 7. *Mycoplasma* spp. and *Ureaplasma* spp.

### 7.1. Epidemiology

Mycoplasma is a term used to refer to any of the members of the class *Mollicutes*, which includes *Mycoplasma* and *Ureaplasma*. Genital mycoplasmas associated with human infections include *Ureaplasma urealyticum* (*U. urealyticum*), *Ureaplasma parvum* (*U. parvum*), *Mycoplasma hominis* (*M. hominis*), and *Mycoplasma genitalium* (*M. genitalium*). These STIs are not notifiable, and few data exist on their prevalence. However, *Mycoplasma* spp. and *Ureaplasma* spp. are considered natural inhabitants, as they are often isolated from healthy individuals [109]. Regarding *M. genitalium*, recent studies reported a prevalence ranging between 1% and 3.3% in the general population of both sexes [112].

European studies have reported overall prevalence in males (aged 15–79 years) and females (aged 16–65 years) ranging from 4.9% to 9.8% [113]. As expected, population-based prevalence estimates are lower than those derived from clinic-based studies, which have reported rates as high as 26% in women [114] and 28.7% in men attending STIs clinics [115]. *M. genitalium* has also been detected in the cervix or endometrium of women with pelvic inflammatory disease, with prevalence estimates ranging from 4% to 22% [41].

Recently, an Italian study reported an overall prevalence of 7% for *M. genitalium* in the general population, with the highest infection rates found in younger age groups as follows: 9.5% in the 13–18 age group and 6.7% in the 19–29 age group [3].

The epidemiology of *M. hominis*, *U. parvum*, and *U. urealyticum* has been described in various reports worldwide, with a prevalence of 21% for *Ureaplasma* spp. and 3% for *M. hominis* in the general population [116]. *M. hominis*, a commensal of the cervical and vaginal mucosa, exhibits colonization rates ranging from 20% to 30% globally [117].

In Italy, a recent study highlighted the prevalence of *M. hominis* and *Ureaplasma* spp., reporting an overall *M. hominis* prevalence of 11.4%, with marked sex differences (4.1% in men and 21.1% in women). Younger age groups exhibited higher *M. hominis* infection rates, with a prevalence of 23.8% in individuals aged 13–18 years and 13.7% in those aged 19–29 years. Similarly, the prevalence of *U. urealyticum* increased post-pandemic, with a prevalence of 19.3% among female patients. Notably, the age group 13–18 years showed a prevalence of 14.3%, compared to 15.7% in the 19–29 age group. The overall prevalence of *U. parvum* was 26.1%, with particularly high rates of 52.1% in females and 57.1% in the 13–18 age group [3].

### 7.2. Clinical Manifestations

*M. genitalium* is responsible for 15–20% of non-gonococcal urethritis (NGU) in men, 20–25% of non-chlamydial NGU, and 40% of persistent or recurrent urethritis [114,118,119]. Asymptomatic infections are common, occurring at similar rates as chlamydial infections. In women, *M. genitalium* is linked to cervicitis, PID, preterm delivery, spontaneous abortion, and infertility, with potential mother-to-child transmission at birth. However, there is insufficient evidence to suggest that *M. genitalium* causes chronic complications in men, such as epididymitis, prostatitis, or infertility. Rectal infections are documented in 1–26% of MSM and 3% of women. Persistent or recurrent infections require accurate diagnosis and treatment [120,121].

*M. hominis*, a commensal organism of the lower urogenital tract, can cause a range of infections, including urinary tract infections (UTIs), bacterial vaginosis, PID, cervicitis, and pyelonephritis. It is also associated with pregnancy complications, infant disease, and infertility [122,123]. A clinical association with *Trichomonas vaginalis* has been observed [124]. *Ureaplasma species*, another group of potentially pathogenic microorganisms, contribute to NGU, infertility, and uterine inflammation. These microorganisms can ascend the reproductive tract and stimulate immune responses, leading to higher levels of proinflammatory cytokines in the cervicovaginal environment [125,126,127]. *Ureaplasma* infections are linked to adverse pregnancy outcomes such as low birth weight, chorioamnionitis, premature rupture of membranes, spontaneous abortion, and perinatal or neonatal death [128].

### 7.3. Testing and Screening

NAATs are the gold standard technique for testing urogenital *Mycoplasma* spp. and *Ureaplasma* spp. Although culture is not useful for diagnostic purposes, it plays a role in obtaining isolates for antimicrobial resistance testing and research [129,130]. Serology testing for antibodies, unfortunately, is affected by cross-reactivity with other mycoplasmas, including *Mycoplasma pneumoniae* [118]. Syndromic STI panels using multiplex PCR for CT, NG, TV, *M. hominis*, *M. genitalium*, *U. parvum*, and *U. urealyticum* are more commonly used for detection and clinical diagnosis. These tests have been developed using various amplification and detection techniques, gene targets, and platforms, with samples from liquid-based cytology, urine, genital swabs, oropharyngeal swabs, anorectal swabs, and semen. Current guidelines [38] suggest the use of multiplexed technologies for STIs; it is the method with the best performance compared to isolation and permits the identification of multiple pathogens simultaneously. The IUSTI Europe 2021 guidelines recommend the use of real-time PCR testing to detect *M. genitalium* and simultaneously identify macrolide resistance [38].

The CDC do not recommend asymptomatic screening for *M. genitalium*, as there is no evidence that asymptomatic infection requires treatment [129,130]. European guidelines recommend testing only for symptomatic individuals such as those with symptoms of NGU in men and pelvic inflammatory disease in women [119].

### 7.4. Treatment

β-lactams, including penicillins and cephalosporins, are ineffective against *M. genitalium* because it lacks a cell wall. Resistance to azithromycin is increasing rapidly, so resistance-guided therapy has been shown to achieve cure rates of more than 90% and should be used whenever possible [131,132]. If *M. genitalium* resistance testing is available and the bacteria is sensitive to macrolides, the recommended therapy is as follows: doxycycline 100 mg orally two times/day for 7 days, followed by azithromycin 1 g orally initial dose, followed by 500 mg orally daily for 3 additional days (2.5 g total) [41]. This dosage is recommended for the treatment of cervicitis in non-pregnant women [38]. For the treatment of male urethritis, the first-line treatment is the administration of azithromycin or josamycin or moxifloxacin. Azithromycin is administered orally at a dosage of 500 mg on the first day and 250 mg for 2–5 days, while josamycin is administered orally at a dosage of 500 mg every 8 h for a duration of 10 days. Moxifloxacin is administered orally, 400 mg for 7 days. If resistance testing is not available and *M. genitalium* is detected by an FDA-approved NAAT, treatment involves doxycycline 100 mg orally two times/day for 7 days, followed by moxifloxacin 400 mg orally once daily for 7 days. For male urethritis, if treatment with azithromycin proves ineffective, the second-line drug is moxifloxacin, administered as a single oral dose of 400 mg for a period of seven days. If both azithromycin and moxifloxacin prove insufficient, the infection may be treated with oral administration of pristinamycin (1 g every 6 h for 10 days), minocycline (100 mg every 12 h for 14 days), or doxycycline (100 mg every 12 h for 14 days) [38,41].

Given the reported cases of moxifloxacin resistance, adverse side effects, and cost, when resistance testing is not available or when moxifloxacin cannot be used, an alternative regimen includes doxycycline 100 mg orally twice a day for 7 days, followed by azithromycin (1 g orally on day 1, followed by 500 mg once daily for 3 days). In this case, the guidelines recommend a follow-up test 21 days after the completion of therapy [41].

Treatment of *M. genitalium* during pregnancy should be considered because of the potential teratogenicity of the recommended first-line drugs. Consultation with the National Network of STD Prevention Training Centers STD Clinical Consultation Network is therefore recommended [133].

There are no international evidence-based management guidelines for *M. hominis*, *U. parvum*, and *U. urealyticum*, and there is a lack of evidence regarding effective treatment regimens. Like *M. genitalium*, *M. hominis* is not treatable with ß-lactam antibiotics. *M. hominis* is also naturally resistant to azithromycin, clarithromycin, and erythromycin (14- and 15-membered macrolides), but not to josamycin (16-membered macrolides). Antimicrobial resistance is not the only obstacle to treating *M. hominis*, *U. parvum*, and *U. urealyticum*. These organisms can also be difficult to eradicate due to the reduced activity of antimicrobials at low pH and the lack of bactericidal activity [134,135].

Nevertheless, the data collected by AMCLI propose first-line and second-line treatments for the management of male urethritis caused by *U. urealyticum*. If oral administration of 100 mg of doxycycline every 12 h for 7 days does not successfully resolve the infection, the patient may be treated with a single oral dose of 1 g of azithromycin [39].

## 8. Antimicrobial Resistance (AMR)

### 8.1. Neisseria gonorrhoeae

Recently, the European Centre for Disease Prevention and Control (ECDC) has published a report highlighting the threat of increasing antimicrobial resistance (AMR) in *Neisseria gonorrhoeae*. This concerning trend, identified from surveillance data, underscores the need for continuous monitoring to guide and update treatment guidelines, control measures, and to ensure the prudent use of antimicrobials, in the context of rising gonorrhea cases in Europe [136]. The main classes of antibiotics involved include extended-spectrum cephalosporins (such as ceftriaxone and cefixime), azithromycin, ciprofloxacin, and penicillin. Generally, clinical manifestations do not differ significantly between resistant and non-resistant strains; however, infections caused by resistant strains may result in persistent or recurrent symptoms.

Specific resistant strains have been identified, such as strain FC428, which was first isolated in Japan in 2015 and shows resistance to ceftriaxone [137]. Similar strains have been identified in several countries, including China and the United States [137,138]. Following that, strains SS43 and SS76 were isolated in Guangzhou, China, in 2016 and 2019, respectively. These strains exhibit resistance to ceftriaxone and azithromycin, as well as resistance to penicillin, tetracyclines, and ciprofloxacin [92]. Meanwhile, strain ST1407 has been identified in various countries, including France, Spain, Argentina, and Brazil. It is associated with treatment failures and shows resistance to ceftriaxone and azithromycin [139]. Moreover, studies have documented the spread of *N. gonorrhoeae* strains resistant to multiple classes of antibiotics, known as MDR (multidrug-resistant) strains [140].

Surveillance data on gonococcal antimicrobial susceptibility in the European Union/European Economic Area for 2022 show, in 2022, the percentage of strains resistant to azithromycin significantly increased to 25.6%, compared to 14.2% in 2021. Azithromycin is often used in combination with ceftriaxone to treat gonorrhea, making this result particularly concerning. Ciprofloxacin resistance has also increased, with 65.9% of isolated strains showing resistance in 2022, compared to 62.8% in 2021 [136].

Recently, a frequent detection of resistance to ciprofloxacin was observed among *N. gonorrhoeae*-positive subjects, not only in those older than 40 years, but also among younger people [141].

Although resistance to cefixime remains low at 0.3%, continuous monitoring is crucial, particularly as cefixime- and ceftriaxone-resistant gonococcal strains are spreading internationally.

At a molecular level, *N. gonorrhoeae* has acquired antimicrobial resistance (AMR) through various mechanisms, including the enzymatic alteration or breakdown of the antimicrobial, shielding of the antimicrobial target, and reduced uptake combined with enhanced expulsion of the antimicrobial [137,141]. *N. gonorrhoeae* resistance to extended-spectrum cephalosporins (ESCs), such as ceftriaxone and cefixime, is primarily due to mutations in the penA gene, which codes for altered versions of penicillin-binding protein 2 (PBP2). Along with penA mosaic alleles, other genetic variations in the mtrR, porB1b, and ponA genes contribute significantly to the decreased susceptibility to cefixime and ceftriaxone [142]. These include the overproduction of the MtrCDE efflux pump system, reduced membrane permeability, and changes in the ponA gene. Fluoroquinolone resistance is caused by mutations in the A subunit (codons Ser91 and Ser95) of DNA gyrase (gyrA) and in the ParC subunit of topoisomerase IV (parC). Point mutations in the peptidyl-transferase loop region of domain V of 23S rRNA, along with mutations in ribosomal proteins L4 (rplD) and L22 (rplV), are responsible for macrolide resistance, such as azithromycin [142,143,144].

### 8.2. Mycoplasma genitalium

The absence of a bacterial cell wall in mycoplasmas restricts the range of effective antimicrobial classes, including tetracyclines, macrolides, fluoroquinolones, and streptogramins [145]. Additionally, resistance to key treatment classes, such as macrolides and fluoroquinolones, is on the rise, jeopardizing the ability to effectively treat all infections. Antimicrobial resistance in *M. genitalium* is becoming an increasingly serious issue. The prevalence of reported macrolide resistance varies significantly across different regions and countries. Over 40% of *M. genitalium* strains are currently resistant to macrolides, which have traditionally been considered the first-line treatment for this type of infection. It is believed that the occurrence of MRM is more common in individuals who undergo repeated courses of azithromycin or other macrolide antibiotics [146]. Consequently, men who have sex with men (MSM) generally show a higher rate of MRM compared to women or men who have sex with women, likely due to more frequent treatment for *M. genitalium*, chlamydia, and gonorrhea [131]. High rates of macrolide resistance in *M. genitalium* have been reported in Europe (especially the UK and Denmark) and Australia [147]. Approximately 15–30% of strains are also resistant to the second-line therapy, fluoroquinolones, especially moxifloxacin [145], and it is particularly significant in Japan [148].

At the molecular level, macrolide resistance is caused by single-nucleotide polymorphisms (SNPs) in the 23S ribosomal RNA (rRNA) gene at positions A2058 or A2059 [131]. Resistance to the second-line drug moxifloxacin is driven by SNPs in the topoisomerase IV gene parC, which result in amino acid alterations in the quinolone resistance-determining region, mainly at parC positions S83 and D87 (according to *M. genitalium* amino acid numbering). These mutations associated with quinolone resistance (QRAMs) are becoming more prevalent globally [131].

## 9. Discussion

Recent epidemiological data have highlighted a concerning worldwide increase in sexually transmitted infections, particularly among adolescents and young adults. Factors such as age, gender, sexual debut, and sexual orientation play a significant role in the epidemiology of STIs, with young people and adolescents being particularly vulnerable. The prevalence of infections has also increased among young heterosexuals, marking a shift in lifestyle patterns, where gaps in sexual knowledge, attitudes, and behaviors have emerged and may have potentially contributed to the rise in new infections. The most frequent barrier in the management of STIs is the lack or fragmentation of the local healthcare services [10]. This aspect is particularly relevant for young people and adolescents. The message of this review highlights the need for a permanent multidisciplinary healthcare system based on an integrated clinical and sexual educational network able to ensure effective interventions. In particular, collaboration between STI healthcare services and schools, designated places for equitable health promotion through a continuous educational approach, is essential [8]. For these reasons, national programs funded by the Italian Ministry of Health have focused on developing a multidisciplinary model for STIs [11,12,149,150].

These initiatives include the implementation of educational activities in schools, addressing sexuality, emotional relationships, and STI prevention [137], including screening that facilitates real-time detection and treatment of infections. In this context, routine personalized screening for asymptomatic infections, such as gonorrhea and chlamydia, could be recommended at least annually for adolescents [150,151,152]. Nevertheless, self-testing kits, mobile clinics, and community outreach programs, recently proposed for at-risk populations, could help to increase access to healthcare services if included in the local STI network [153].

## 10. Conclusions

In conclusion, public health initiatives should prioritize the development of multidisciplinary networks specifically relating to the needs of adolescents through a comprehensive approach that integrates education and clinical support to tackle the rising prevalence of STIs and to raise awareness of safe sex.

## Data Availability

No new data were created or analyzed in this study. Data sharing is not applicable to this article.
